# Growth factor thermostability dataset derived from experimental and computational approaches to accelerate cultured meat development

**DOI:** 10.1016/j.dib.2026.113042

**Published:** 2026-06-27

**Authors:** Wei Ng, Kiana Aghakasiri, Rebecca Pierce, Julia Marschallinger, Kimberly J. Ong, Steven D. Rees, David Staszak, Breanna Duffy

**Affiliations:** a Vireo Advisors, LLC, Boston, Massachusetts, USA; b University of Alberta, Edmonton, Alberta, Canada; c Alberta Machine Intelligence Institute, Edmonton, Alberta, Canada; d Defined Bioscience, Inc, San Diego, California, USA; e New Harvest, Sacramento, California, USA

**Keywords:** Protein stability, Thermal shift assay, FoldX, Large language model, Recombinant proteins, Cellular agriculture, Cultured meat safety

## Abstract

This project compiled experimentally measured and computationally predicted thermostability data for growth factors (GFs) commonly used in cultured meat and seafood (CM) production. Data were generated on recombinant GFs using thermal shift assays and combined with publicly available data using a structured literature curation workflow developed using a Large Language Model (LLM) with manual validation. In addition, *in silico* protein stability calculations were generated using FoldX. Data such as GF identity, species origin, recombinant host, variant information, method of measurement, and experimental conditions were collected and standardized into a tabular format. The combined dataset contains 513 melting temperature (Tm) values across diverse GFs and variants, along with additional thermodynamic parameters on a subset of 15 GFs.

The data were harmonized, standardized, and released as an open-source dataset suitable for computational modeling, machine learning workflows, culture media design, and safety assessment frameworks. This resource supports research evaluating GF stability under production- and processing- relevant conditions and provides a foundation for future data expansion, including additional GF variants and other growth factors. By consolidating dispersed thermostability measurements into a unified dataset, supported by experimental data, the resource supports accessibility and reuse across the CM, protein engineering, and regulatory science communities.

Specifications Table

**Table:** 

Subject	Biological Sciences
Specific subject area	Thermostability data of growth factors generated through experimental and computational approaches
Type of data	Table, Graph Processed
Data collection	Thermal shift assays were performed on recombinant growth factors using the ThermoFisher Protein Thermal Shift dye and the Applied Biosystems StepOnePlus RT-PCR system. *In silico* protein stability features were generated using FoldX software on published Protein Data Bank structures. Literature thermostability data were extracted and curated using a Large Language Model toolset combined with manual validation and normalization to standardized formats. Inclusion criteria required explicit melting temperatures, and data were harmonized across experimental conditions.
Data source location	Thermal shift assays and FoldX analysis were performed at Defined Bioscience, Inc, San Diego, CA, USA. The literature review was performed using Google Scholar and extracted at Alberta Machine Intelligence Institute, Edmonton, Alberta, Canada and University of Alberta, Edmonton, Alberta, Canada.
Data accessibility	Repository name: Growth factor thermostability dataset derived from experimental and computational approaches to accelerate cultured meat development Data identification number: 10.5281/zenodo.19339684 Direct URL to data: https://zenodo.org/records/19339684
Related research article	none

## 1 Value of the Data

• Culture media development and safety evaluation for cultured meat and seafood (CM) production are limited by the paucity of public data on growth factor (GF) thermostability and other safety characteristics. To this end, we developed an open-source dataset containing GF thermostability data from the public domain, experimental data from thermal shift assays, and *in silico* data on protein thermodynamic features.
• The dataset is open-source and is intended to inform the development of food-safe recombinant GFs, support computational workflows for predicting GF stability, and guide CM safety evaluations. Thermostability data help determine whether residual GFs are degraded during heat processing or cooking, which is relevant when GF levels in harvested cell material or the final food product are detected and require further safety evaluation.
• The dataset provides a foundation for sharing pre-competitive safety data and promoting transparency in food risk assessments and policy-making processes. Because these measurements are not company-specific know-how, they demonstrate a type of pre-competitive dataset that could be openly shared by the field to reduce duplicated testing and improve comparability across CM development efforts.
• As engineered GFs and GFs from diverse species become more common in CM production, the dataset could be expanded to incorporate safety data on additional GFs.

## 2 Background

Cultured meat and seafood (CM) aims to produce complementary animal proteins from cell cultures [1]. While many CM ingredients have a history of safe use in food, growth factors (GFs) are essential manufacturing inputs without historical use as food additives or processing aids [[1], [2], [3]]. GF stability during manufacturing (i.e., physiological temperatures) is necessary for regulating cell growth and differentiation, while denaturation during food processing (e.g., cooking temperatures) can reduce food safety risks [2,4], which has been considered in CM safety regulatory reviews [5,6]. Computational tools could provide insights into GF thermostability, particularly as innovators optimize GFs for CM production. However, models require significant amounts of data, and many available models rely on theoretical thermostability [7]. Currently, public data on GF thermostability are scarce and confined to individual journal publications.

We developed an open-source dataset of GF thermostability data from existing literature and new measurements. A custom Large Language Model (LLM)-based toolset was developed to aggregate data from existing literature, additional data was generated through thermal shift assays, and *in silico* data on protein features were generated using FoldX. This dataset is intended to inform the development of GFs, support the application of computational workflows, and guide culture media safety evaluations.

## 3 Data Description

This work includes data derived from 3 sources (*in vitro* thermal shift assays, *in silico* FoldX-generated protein features, and thermostability data from prior literature), which were combined into a publicly available dataset. This section describes each data source individually before describing the final dataset.

### 3.1 *In vitro* analysis of select growth factors using thermal shift assays

**Fig. 1:**
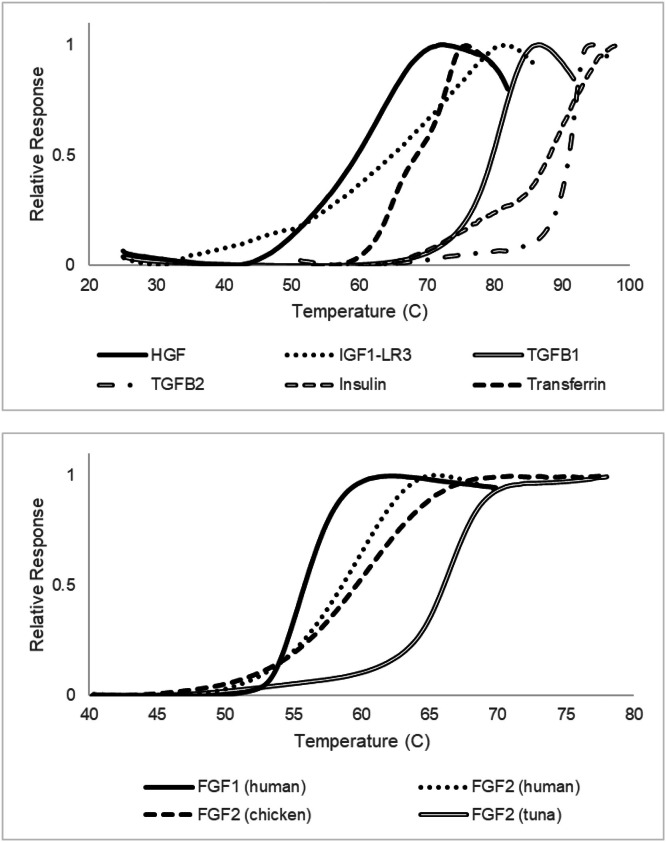
Thermal shift assay melting curves for the recombinant growth factors analyzed in this dataset. Normalized fluorescence is plotted as a function of temperature.

Thermal shift assays were performed on recombinant GFs frequently used in CM production. Melting curves were successfully obtained for 10 proteins after 2-4 assay trials (Fig. 1). Melting temperature (Tm) values ranged from 55.7°C (FGF1) to 91.0°C (TGFB2) (Table 1). Insulin (88.1°C), TGFB1 (79.9°C), and TGFB2 (91.0°C) showed higher Tm values, while transferrin (68.6°C), HGF (59.6°C), and IGF1-LR3 (65.0°C) exhibited lower Tm values (59-69°C). Within the FGF family, human FGF2 was less thermostable (58.6°C) than tuna FGF2 (66.0°C) under these assay conditions.

**Table 1:** Melting temperatures (Tm) of growth factors determined by thermal shift assays.

Protein	Species	Tm (°C)
FGF1	human	55.7
FGF2	human	58.6
FGF2	chicken	59.5
FGF2	tuna	66.0
HGF	human	59.6
Insulin	human	88.1
IGF1-LR3	human	65.0
TGFB1	human	79.9
TGFB2	human	91.0
Transferrin	human	68.6

Abbreviations: fibroblast growth factor (FGF); transforming growth factor beta (TGFB), hepatocyte growth factor (HGF); insulin-like growth factor 1 long R3 analog (IGF1-LR3).

### 3.2 *In silico* analysis of growth factors using FoldX

*In silico* stability analysis was performed for select GFs using FoldX to calculate folding free energy (ΔG) values based on available Protein Data Bank (PDB) structures. 47 PDB structures of 14 relevant GFs were included. All calculated ΔG values, corresponding PDB identifiers, and FoldX output files are provided in the “FoldX Data” tab of the associated data repository [6].

FGF2 consistently showed tight clustering across structures (ΔG values -6.2 to -1.2). Similarly, Insulin displayed consistent ΔG values across different PDB entries (6.4-7.8). For FGF1, ΔG values varied across different PDB entries, spanning from −2.8 to 24.4, depending on the structure analyzed. High variation was also observed for transferrin (−12.8 vs. 119.8). Within the TGF/BMP superfamily, TGFB1 had the highest ΔG (57.3), while TGFB2 and TGFB3 showed intermediate values (12.1-21.5). BMP-2 (used as a surrogate for BMP-4; 88% sequence identity) yielded a wide range of ΔG values (0.3-31.6). These wide ranges exemplify how FoldX-estimated ΔG values vary across PDB structures due to differences in resolution, protein context, and full complex assembly (see EXPERIMENTAL DESIGN, MATERIALS AND METHODS). Many growth factors here are natively oligomeric. For BMP-2 specifically, interface energies calculated by FoldX AnalyseComplex were consistent across structures (-31 to -35 kcal/mol) despite the total ΔG ranging from 0.3 to 31.6 kcal/mol.

Absolute ΔG values should be treated qualitatively; comparative ΔΔG values are recommended to assess the impact of mutations, which is beyond the scope of this work.

### 3.3 LLM-extraction of thermostability data from literature

We conducted a literature review to identify and collect thermostability data on GFs from previously published studies. Large Language Models (LLMs) were utilized to expedite the literature review process, as well as manual validation and additions. In total, 503 Tm values were extracted from the literature across 42 publications. These data cover a range of 33 growth factors across 16 species, including a variety of growth factor variants which lead to changes in Tm. Tm values from the literature were measured using a variety of methods, primarily including Circular Dichroism, Differential Scanning Calorimetry, Fluorescence Spectroscopy, and Thermal Shift assay.

### 3.4 Growth factor thermostability dataset

The GF thermostability data from literature and from *in vitro* and *in silico* analyses conducted as part of this work were collated into a unified dataset and released for open source use. The final dataset includes 513 melting temperatures for 32 unique GFs and their variants spanning 42 species orthologs, as well as thermodynamic parameters for 47 structures of 15 GFs. The dataset includes information on GF identity, species/biological origin, sequence modifications, and host organism (if recombinantly produced). For experimental data, details on experimental conditions, including pH and buffer, salt, and protein concentrations, were also provided, where available. The following parameters from the FoldX analysis, which may contribute to predictions of protein stability in future computational workflows, were also included in the dataset alongside protein structure PDB identifiers: total energy, backbone H-bond, sidechain H-bond, Van der Waals, electrostatics, solvation (polar), solvation (hydrophobic), Van der Waals clashes, entropy side chain, entropy main chain, cis bond, torsional clash, helix dipole, disulfide, and energy Ionisation. Due to the distinct, but complementary, nature of the two types of data, the experimentally determined melting temperatures and FoldX-derived thermodynamic parameters are included in separate tables of the dataset. The first 3 columns of both tables are consistent, allowing users to merge the data if desired. The dataset can be accessed here: https://zenodo.org/records/19339684 [8].

## 4 Experimental Design, Materials and Methods

### 4.1 Thermal Shift assay (TSA)

GFs commonly used in CM production were purchased from the following suppliers: Insulin (ThermoFisher, A11382IJ), Transferrin (InVitria, 777TRF029), FGF2 (Qkine, Qk027), FGF2 (G. gallus) (Defined Bioscience, LSR-202), FGF2 (T. albacares) (Defined Bioscience, LSR-120), TGFB1 (Qkine, Qk111), IGF1-LR3 (Qkine, Qk041), FGF1 (Qkine, Qk071), TGFB2 (Qkine, Qk072), HGF (Qkine, Qk013). Unless otherwise noted, proteins were recombinant human. Purified GFs were prepared at concentrations of 0.25-1 mg/mL in the manufacturer-recommended buffer as follows:

• Insulin, transferrin, FGF2 (T. albacares), FGF2 (G. gallus): 20 mM KPO4 pH 7.5, 500 mM NaCl
• TGFB1, TGFB2, HGF: 10 mM HCl
• IGF1-LR3: water (reconstituted from lyophilization in TFA/ACN, concentrations not disclosed by manufacturer)
• FGF1 (human): water (reconstituted from lyophilization in Tris, NaCl, and CyS; concentrations not disclosed by manufacturer)
• FGF2 (human): water (reconstituted from lyophilization in Tris, NaCl, CyS, and mannitol; concentrations not disclosed by manufacturer)

Thermal shift assays were used to generate experimentally determined Tm for individual GFs. Measurements were performed using the Thermo Protein Thermal Shift (PTS) dye (Thermo Fisher, Cat. no. 4461146), which emits fluorescence upon binding to hydrophobic regions exposed during protein unfolding. Each protein was diluted and mixed with PTS according to manufacturer instructions, and loaded into a 96-well plate compatible with the Applied Biosystems StepOnePlus RT-PCR system. The temperature was increased from 25°C to 99°C at a rate of 1°C per step. Fluorescence intensity values were normalized prior to curve fitting, and Tm for each protein was determined by analyzing the derivative of the fluorescence data to identify the inflection point. Each sample was analyzed in quadruplicate, with buffer-only samples and known denatured proteins as controls included for validation. Tm values were averaged from quadruplicate measurements and reported with standard deviations.

### 4.2 FoldX analysis

The intrinsic stability of 14 GFs relevant to cultured meat production (FGF1, FGF2, FGF6, IGF1, NRG1-B1, insulin, HGF, EGF, ovotransferrin, transferrin, TGFB1, TGFB2, TGFB3, BMP-4) was assessed using FoldX software (version 4) [9]. FoldX is an empirical force-field-based program that estimates protein folding free energy (ΔG; kcal/mol) from three-dimensional structures [10]. Per-structure folding free energy (ΔG) and individual energy contribution terms (e.g., hydrogen bonding, van der Waals, solvation, entropy) were calculated. For each entry, the “Total Energy, ΔG” output corresponds to the predicted protein folding free energy as defined by the FoldX energy function. ΔG values are used in a comparative, rather than absolute, manner.

For each protein, one or more experimental structures were selected from the Protein Data Bank (PDB) that best represented the biologically relevant form (monomer or dimer), had a high resolution (≤ 2.5 Å, or the best available), species matching the commercial protein, and complete domain coverage. Where possible, model completeness, R/Rfree values, chain coverage, and absence of unresolved regions were taken into account. In cases where no experimental structure was available, either a homologous experimental structure was used (*e.g.* BMP-2 used as surrogate for BMP-4, 88% protein sequence identity), or a high-confidence AlphaFold-predicted model (*e.g*. FGF-6). The results from these homologous structures should be viewed as qualitative approximations for structural context rather than quantitative predictions of Tm.

Structures were preprocessed using the FoldX *RepairPDB* command to correct geometry and add missing atoms. Stability calculations were then performed with the *Stability* command at 298 K under default FoldX parameters, and mean ΔG ± SD values were reported. For proteins with multiple available structures, each was analyzed independently, and results were reported per structure. Values were not averaged across entries due to observed structural variation. Energy-term outputs and mean ΔG values are provided in the associated data repository [8].

Structure-specific considerations: Differences in absolute FoldX "total energy" values across structures should not be interpreted as direct measures of intrinsic protein stability. FoldX total energies are strongly influenced by structure-specific factors, including experimental resolution, construct boundaries, missing residues, crystal packing effects, and local geometric strain. Different structures of the same protein can yield substantially different total energies despite representing the same folded state. Positive total energies do not imply unfolded proteins; they reflect balance of terms relative to FoldX's reference state, particularly for monomers extracted from oligomeric structures.

### 4.3 LLM-guided literature review

Literature evaluation focused on GFs commonly used in the production of CM, including BMP-4, EGF, FGF-2, HGF, IGF-1, IGF-1-LR3, insulin, transferrin, IGF-2, IL-4, IL-6, TGF-β1, TGF-β2, and TGF-β3. Data on additional proteins (e.g., other FGFs, hemoglobin, lactoferrin, albumin) were included in the dataset if identified in the literature search. The search was performed using Google Scholar and defined search terms, including “thermostability”, “heat degradation”, “melting temperature”, and/or “thermal stability”, and common synonyms for GF names (see Supplemental files). Thermostability data were lacking or limited for some GFs (BMP-4, IGF-1, IGF-2, IL-4, TGF-β3). Studies that did not report a Tm value or reported data already presented in another source were excluded. Relevant literature was downloaded as PDF files, including all supplementary files.

A custom LLM-based toolset was developed to assist with data extraction from identified publications. Data extracted from publications include GF identity, species/biological origin, host organism (if recombinantly produced), description of any sequence modifications, details on experimental method (e.g., thermal shift assay, circular dichroism), and conditions (e.g., protein concentration, pH, buffer conditions) for evaluating GF stability and Tm.

To extract and aggregate the existing data, we employed a multi-stage data extraction approach, pulling groups of data elements out of the publications incrementally and including rounds of manual review and other consistency checks to ensure a reliable dataset. Models using OpenAI (gpt 03-mini, GPT 4.1) [11], Google (Gemini 2.5 Pro) [12], and Deepseek (r1) [13] were tested and compared to extract the initial data elements (including Author/Year of Publication, Paper Title, Species/Biological Origin, Variant, and Tm) from a ground truth sample of six publications. After settling on the most performant model (DeepSeek r1), we then extracted these data elements from the whole of the identified publications. Since some publications report multiple Tms (including for a single protein or for multiple proteins), we chose to assign a single row in the table to unique combinations of publication, protein (including wild-type or variant), species, and melting temperature. From this, we found and removed the subset of papers that did not explicitly report a melting temperature (e.g., reported a range or contained a description of protein thermostability). Melting temperature values from the remaining papers were manually validated.

Table 2 shows the accuracy of Tm extraction by the LLM for papers that report a melting temperature. Of the 321 rows generated by the LLM from 38 papers, 292 (91.0%) were correct. Of the 29 incorrect values, 6 (1.9%) were cases where the Tm value existed in the paper, but the reported value was incorrect; 19 (5.9%) were cases where a value did not exist in the paper and a value was falsely generated; and 4 (1.2%) lacked a specific numeric Tm value (Table 2). Additionally, the model missed 95 additional Tms found in manual review, resulting in a total of 393 validated rows with data extracted by the LLM in the final dataset.

**Table 2:** Accuracy of Tm identification.

Data Element	Tms in LLM-generated dataset (n=321)				Tms in LLM-generated dataset (n=321)	
	Correct	Incorrect^a^	Falsely Generated^b^	Lacked a Specific Numeric Value^c^	Correct	Incorrect^d^
Tm	292 (91.0%)	6 (1.9%)	19 (5.9%)	4 (1.2%)	26 (68.4%)	12 (31.6%)

a LLM reported a value from the paper that did not correspond to the correct Tm.

b LLM reported a value that could not be found in the paper.

c LLM did not report a specific numeric Tm value (e.g., listed as 'Not determined' or similar).

d Papers with any incorrectly reported or falsely generated Tms, or Tms that lacked a specific numeric value (e.g., listed as 'Not determined' or similar) were categorized as incorrect.

Each additional data element/column for the table was extracted with a separate prompt. For this data extraction, GPT 4.1 was found to be most performant on the ground truth data due to its long context window (1M tokens), which was particularly effective at handling longer articles, such as extensive supplementary materials, theses, and dissertations. For more challenging elements, like GF and production organism, we included a list of possible values in the prompt as examples to help guide the model (i.e., few-shot prompting). For Amino Acid Sequence information, we extracted the UniProt ID and any explicitly mentioned variants, since full sequences were often missing or inconsistently formatted across papers. Additionally, we integrated explicit citations of section/page for the data into the model’s outputs to simplify the subsequent manual validation process.

For consistency, we utilized GPT-o4-mini as a judge to perform checks on columns that shared common information across entries (including Experimental Condition with Buffer, PH, and Protein Concentration elements). Additionally, we developed and applied regex-based scripts to flag potentially inconsistent data points, which were then manually verified and corrected (including Tm, Amino Acid Sequence, and Engineered Variant elements). Finally, a manual review was performed to validate the key data elements. The automated checks helped guide the manual verification process by highlighting rows that were likely problematic. However, the manual review went beyond just the flagged case, and the final corrections relied heavily on careful row-by-row review of the original source content.

We performed a performance estimate of the accuracy of the LLM extractions of the Uniprot, Species Origin, pH, Protein Concentration, and Buffer columns. The analysis compared the raw output of the LLM to the human-verified values in the final dataset. Table 3 shows the accuracy of extraction for these additional columns. For each of the 393 rows (from 38 unique papers), we show the fraction that the LLM correctly extracted and the fraction that it got wrong (either ‘Falsely Generated’ or ‘Incorrect’) by each column.

**Table 3:** Accuracy of additional data columns.

Data Element	Tms in final dataset (n=393)			Papers extracted by LLM (n=38)		
	LLM Correct (n, rows)	LLM Incorrect^a^ (n, rows)	Fraction Correct (%, rows)	LLM Correct (n, papers)	LLM Incorrect^b^ (n, papers)	Fraction Correct (%, papers)
Uniprot ID	385	8	98.0%	37	1	97.4%
Species Origin	390	3	99.2%	37	1	97.4%
pH	354	39	90.1%	35	3	92.1%
Protein Concentration	379	14	96.4%	36	2	94.7%
Production Organism	243	150	61.8%	28	10	73.7%
Buffer	336	57	85.5%	32	6	84.2%

a LLM reported value was incorrect in any way for the specific row. Differences in nomenclature (e.g., E. coli vs Escherichia coli, human vs Homo sapiens) were considered correct.

b Papers with any incorrectly reported or falsely generated values were categorized as incorrect.

The LLM performed especially well for simpler tasks, such as Uniprot ID (98.0% correct rows, 97.4% correct papers), Species Origin (99.2% correct rows, 97.4% correct papers), pH (90.1% correct rows, 92.1% correct papers), and Protein Concentration (96.4% correct rows, 94.7% correct papers). The LLM performance was lower for Production Organism (61.8% correct rows, 73.7% correct papers) and Buffer (85.5% correct rows, 84.2% correct papers). For Production Organism, the LLM failed to recognize when GFs were not produced recombinantly, reporting a false species rather than reporting “not applicable”. Giving the prompts additional context around this particular failure mode would likely improve the results. For Buffer, relevant details were often fragmented within the paper, with information on the basal buffer solution and additives reported across multiple sections (e.g., methods, figure caption/legend, supplemental material), increasing the likelihood of the LLM missing information. The accuracy of buffer component identification, therefore, provides a measure of the model’s ability to recognize and integrate related information regardless of its location within the paper.

All code used for the LLM-guided literature review can be found here: https://github.com/Amii-Applied-AI/amii-cell-ag-tools/tree/main/protein-thermostability-data-tools.

2 additional papers (78 rows) were found after LLM extraction and manually added to the final dataset [14,15]. In addition, 1 paper was replaced with the corresponding thesis, which contained a more comprehensive dataset (32 rows) [16].

### 4.4 Dataset development and open source release

GF thermostability data collected from literature, thermal shift measurements (this work), and data on protein features generated in FoldX were collated into a single dataset [8]. Data and information were manually verified for accuracy and completeness, and terminology (e.g., GF names, species names, description of experimental conditions) was standardized throughout.

## Limitations

• For the thermal shift assay, five GFs (TGFB3, NRG1-B1, IGF1, EGF, and BMP-4) did not yield reproducible melting curves under the initial assay setup and will require protein-specific assay optimization (*e.g.* buffer, pH, salt screening).
• FoldX stability calculations depend on the 3D structure availability and quality. For some GFs, PDB lacked domain coverage or biologically relevant oligomeric states. In these cases, homologous structures or AlphaFold-predicted models were used.
• The inconsistent reporting of Tm and associated data (*e.g.*, GF Uniprot ID, species origin and production organism; experimental conditions) in the literature posed a significant challenge for the LLM extraction pipeline. Variations in the type and placement of data extracted contributed to the observed error rate ranging from 9 - 38%. While this margin was manageable through human curation for a dataset containing several hundred entries, applying this tool to larger datasets would necessitate additional automated validation procedures beyond those developed here.
• The dataset primarily used human protein sequences. These were selected due to their extensive prior characterization and high availability of structural data. As the CM field shifts toward GFs from agriculturally relevant species, the dataset will need to be expanded to capture more thermostability properties of non-human homologs.

## Ethics Statement

The authors have read and follow the ethical requirements for publication in Data in Brief and confirm that the current work does not involve human subjects, animal experiments, or any data collected from social media platforms.

## CRediT authorship contribution statement

**Wei Ng:** Conceptualization, Data curation, Formal analysis, Funding acquisition, Investigation, Project administration. **Kiana Aghakasiri:** Data curation, Investigation, Methodology, Software, Validation. **Rebecca Pierce:** Formal analysis, Investigation, Methodology. **Julia Marschallinger:** Methodology, Project administration, Writing – original draft, Writing – review & editing. **Kimberly J. Ong:** Conceptualization, Investigation, Validation, Writing – original draft, Writing – review & editing. **Steven D. Rees:** Conceptualization, Formal analysis, Investigation, Methodology. **David Staszak:** Conceptualization, Data curation, Formal analysis, Investigation, Methodology, Software. **Breanna Duffy:** Funding acquisition, Project administration, Writing – review & editing.

## Data Availability

ZenodoGrowth factor thermostability dataset derived from experimental and computational approaches to accelerate cultured meat development (Reference data).

## Appendix Supplementary Materials

Image, application 1
